# Review of the Literature on Determinants of Chemical Hazard Information Recall among Workers and Consumers

**DOI:** 10.3390/ijerph13060546

**Published:** 2016-05-31

**Authors:** Farzana Sathar, Mohamed Aqiel Dalvie, Hanna-Andrea Rother

**Affiliations:** Centre for Environmental and Occupational Health Research (CEOHR) and Division of Environmental Health, School of Public Health and Family Medicine, University of Cape Town, Cape Town 7925, South Africa; aqiel.dalvie@uct.ac.za (M.A.D.); andrea.rother@uct.ac.za (H.-A.R.)

**Keywords:** comprehension, memory, recall, warning information, determinants, labels, Globally Harmonized System of Classification and Labelling of Chemicals (GHS)

## Abstract

In many low and middle income countries (LMIC), workers’ and consumers’ only access to risk and hazard information in relation to the chemicals they use or work with is on the chemical label and safety data sheet. Recall of chemical hazard information is vital in order for label warnings and precautionary information to promote effective safety behaviors. A literature review, therefore, was conducted on determinants of chemical hazard information recall among workers and consumers globally. Since comprehension and recall are closely linked, the determinants of both were reviewed. Literature was reviewed from both online and print peer reviewed journals for all study designs and countries. This review indicated that the level of education, previous training and the inclusion of pictograms on the hazard communication material are all factors that contribute to the recall of hazard information. The influence of gender and age on recall is incongruent and remains to be explored. More research is required on the demographic predictors of the recall of hazard information, the effect of design and non-design factors on recall, the effect of training on the recall among low literate populations and the examining of different regions or contexts.

## 1. Introduction

The purpose of a hazard warning is to provide and remind users of relevant hazard information and to promote safety behaviors [1,2]. It is crucial that hazard information for toxic substances be clearly presented and understandable in order to be effective in alerting users of potential hazards and how to safely use the product. Chemical hazard communication is commonly provided in the form of labels and safety data sheets (SDS) [3]. While chemical labels and SDS are accessible to workers, consumers generally only have access to labels. Consumers also tend to use other sources of information for assessing hazards and risks. This was illustrated in a recent study on the risk perception of fracking risks among impacted communities in South Africa, which found that more than 50% of the participants reported media (television, newspapers, radio, internet, magazines, documentaries, and e-mail) and personal experience as sources of health and safety information while less than 25% reported other sources such as friends and family [4]. However, chemicals may have different properties with varying degrees of health and physical hazards, such as carcinogenic, flammable, corrosive, explosive, toxic or harmful to the environment, which is more clearly illustrated on labels. It is important, therefore, for users to understand the potential hazards that are displayed on labels due to high chemical exposure risks both in work and non-work contexts. The Globally Harmonized System of Classification and Labelling of Chemicals (GHS) aims to harmonize chemical hazard communication with the goal of improving comprehension and therefore the effectiveness of the information communicated to workers and consumers [5,6,7]. Comprehension of chemical information, as with health literacy in general, is dependent on the individual’s ability to obtain (*i.e.*, remember and recall) and understand health/hazard information in order to make appropriate risk reduction and health-related decisions. Harmonization of the information contained on labels and SDS is intended to provide consistent information, with the view to promoting better recall and comprehension of chemical hazard information. The GHS also provides a structured system for chemicals sold in Low and Middle Income Countries (LMIC) where a chemical hazard communication system may not be in place to further promote recall and comprehension. Importantly, once a worker or consumer has understood the meaning of chemical hazard information on a label and/or SDS, the key message must be remembered in order to be recalled and applied.

Recall can be defined as the process of retrieving words or pictures from memory [8]. Recall of hazard information, such as the GHS information is crucial for warnings and precautionary information to be effectively understood and applied [8,9]. Failure to recall hazard information during a critical moment when the source of this information is not accessible can likely lead to injury or toxic exposures to a hazard. The recall of information is a cognitive process that is likely to differ between people and for different types of warnings and therefore it is important to understand what factors impact on recall.

Since comprehension and recall are closely linked, the purpose of this literature review is to identify themes as well gaps within the current literature with respect to the comprehension and recall of hazard information. We will explore the comprehension and recall of chemical hazard information among workers and consumers, and synthesize the predictors of comprehension and recall of warning information found in the literature as we hypothesize these impact significantly on recall. The terms comprehension and understanding are used interchangeably in this paper as in the literature.

## 2. Methods

Literature was gathered for this review from both online and print peer reviewed journals. The key databases used for searching literature were EBSCO host via academic search premier, Africa wide information via EBSCO host, Biosis—abstracts, Google Scholar, ScienceDirect, Medline, Scopus and PubMed. The search terms used included (comprehension) AND (memory OR recall) AND (labels OR labeling OR safety data sheets) AND (warning information OR warning design) AND (pictograms OR graphics) AND (demographics OR gender OR age OR education OR training) AND (transport OR industry OR agriculture OR consumer) AND (developed countries OR developing countries) AND (GHS). Data from all study designs and countries were considered.

Less than 10 studies were found that specifically investigated the recall of hazard information generally or the GHS exactly. However, since comprehension and recall are both cognitive processes, we describe the findings of studies that investigated comprehension from which we made inferences about recall. Thereafter, the findings are described of studies that specifically investigated recall. These processes are likely to be connected since comprehension is presented in the literature as enabling a person to recall information [10]. The findings of studies on workers and consumers, which are summarized in Table 1, are presented separately in the text because they are two very different populations. Working with chemicals and other hazards generally presents significantly more dangerous situations that consumers would be exposed to.

### 2.1. Studies on Comprehension of Chemical Hazard Information among Consumers

Previous studies conducted in different settings have shown that the comprehension of hazard information for chemicals is low among both consumers and workers in High Income Countries (HIC) and LMIC [6,11,12,13,14] as most of the participants did not correctly comprehend the majority of the hazard information. In a study of 83 chemistry and biology undergraduate students at Jimma University in Ethiopia, the majority (56.8%) did not understand the hazard warning signs of laboratory chemicals [11]. The low level of comprehension of hazard information among undergraduate students was probably due to the design of the warning sign as they indicated that most of the symbols were difficult to interpret. The participants indicated that they were not guided to pay attention to the warning labels. It should, however, be noted that students may not be comparable to workers since they do not work with hazard information on a daily basis or have received specific training. Lehto (1998) showed a 15 min video about chemical safety and use of labels to engineering students from Purdue University in the United States of America (USA), after which they completed a questionnaire [15]. It was found that the comprehension of labels was correlated with the ease of finding the information on the label (*r* = 0.71). A study conducted by Lesch (2003), in the USA, investigated the impact of training methods on the comprehension of hazard symbols [16]. Participants, recruited through advertisements in local newspapers, were trained to comprehend symbols such as those for “biohazard” or “cancer-causing substance”. The training involved familiarizing the participants with the name of the symbol and a sentence describing the symbol, and then describing to participants an accident scenario relevant to the symbol. When participants were tested after training, comprehension dramatically improved especially among the younger participants aged between 18 and 35 years (88.0% correct) compared to the older participants aged between 50 and 67 years (68.0% correct) [16]. When Lesch (2008) repeated a similar study a few years later, it was found that training improved comprehension, however, there was no difference in comprehension between the younger participants aged between 20 and 35 years (43.0% correct) and older participants aged between 50 and 70 years (41.0% correct) [17]. These studies indicate that training improves comprehension, although the increase in the Lesch (2008) study is far from ideal in a work hazard situation since the majority of the responses were incorrect [17]. It is likely that the effectiveness of training and age on comprehension of hazard information also influences recall of hazard information.

Color blindness and demographic characteristics such as age, gender and level of education has been identified as influencing comprehension of warning information since these may influence cognition [1,2,18]. A survey of four target sectors (agricultural, industrial, transport and consumer) in Zambia found that the level of education, gender and age did not influence the comprehension of GHS label elements, such as the colors, signal words and symbols among consumers [12]. In the latter study, the only means of assessing comprehension was by respondents’ ranking the label elements in the order of the most danger implied (for example, harmful–warning–caution–attention).

### 2.2. Studies on Comprehension of Chemical Hazard Information among Workers

Insufficient training on the use of safety information is a likely factor for poor comprehension of hazard communication among workers. In a study of South African consumers and workers who were regularly exposed to chemicals showed that low levels of comprehension of hazard communication mechanisms such as GHS compliant labels among workers could be due to insufficient training on the use of safety information [6]. In the latter study, about 60% of workers reported that they were trained in occupational health and safety. Three out of twelve warning symbols were found to have more than 50% correct responses among workers, namely, skull and crossbones (81.0%), flammable (61.0%) and explosive symbols (54.0%) [6]. Less than 50% (48%) of the workers in the study reported that they had received training in occupational health and safety and it was not clear if the training was on interpretation of hazard information. In another South African study of 115 farm workers in the Western Cape who were exposed to pesticides, more than half (52.0%) did not know that the pesticide label contained the United Nations Food and Agricultural Organization (FAO) warning and advice pictograms [14]. Of the ten pictograms examined, only one was found to have more than 50% correct responses, namely, wear gloves (74.8%). While a study of 150 Malaysian industrial workers showed a difference in the comprehension of GHS label symbols with the flammable symbol (99.3%) well understood and the compressed gas (27.3%) poorly understood [19]. Similarly, in the South African study on consumers and workers the skull and crossbones (98.0%) and flammable (93.0%) symbols were well understood by workers, whereas the least understood were the corrosive and compressed gas symbols (>5.0%) [6]. Therefore, since these studies found a similar comprehensibility pattern with regards to the most and least understood symbols it is crucial for workers to receive training to improve their comprehension of hazard communication information. The low comprehensibility among workers of most hazard symbols due to a lack of training will most likely impact the recall of hazard information among these workers.

As mentioned before, the study by Banda and Sichilongo (2006) in Zambia found that the level of education, gender and age did not influence the comprehension of GHS label elements, such as the colors, signal words and symbols among workers [12]. Although the latter study (Banda and Sichilongo, 2006) investigated a broad range of users, the demographic characteristics regarding the level of education and age were not clearly presented [12]. In contrast, the study on 150 Malaysian industrial workers showed that a tertiary level education improved the comprehension of GHS symbols compared to those who only completed secondary or primary school [19]. This study also found that a higher position in the workplace led to a better comprehension of GHS symbols, whereas gender and age did not contribute to the comprehension of symbols. However, it must be noted that the majority of the participants were male (92.0%) and between 20 and 49 years of age. In the South African study of 115 Western Cape farm workers, males had more correct responses than females for nine out of the ten FAO pictograms [14]. This was attributed to females associating the pictograms with a social or cultural context since few of the women received training on pesticide safety and what the pictograms actually mean. Therefore, it seems that there is an uncertainty regarding the role of education in determining comprehension and by extension recall of hazard information. These studies also did not find age to influence comprehension of hazard information and the effect of gender is unclear.

### 2.3. Studies on the Recall of Warning Information among Consumers

The study of consumers and workers done in SA also found recall of label elements to be low among both consumers and workers as most of the consumers did not recall most of the label elements [6,20]. A nationwide survey of two thousand randomly selected Ukrainian adults over the age of 18 investigated the role of text warnings on cigarette packs [21]. The sample was reported to represent the demographic and geographic profile of the country. The relationship between recall of warning elements and demographic characteristics was investigated using multivariate analysis. Recall was measured by asking the participants to describe the warnings and was noted as “recalled” if they mentioned specific words on the cigarette packs. This study found that people who completed a higher level of education recalled more warnings. However, recall declined with increase in age. Interestingly, males were more likely to recall warnings, perhaps because they were more likely to smoke.

It has been suggested that pictures are noticed and recalled more easily than words [8,13,18,22]. Pictures are described by Laughery (2006) as design factors that influence the effectiveness of hazard communication tools and will be further discussed in the section on workers but there are a couple of studies that was conducted on consumers [1]. A study of Australian smokers, who were interviewed in four independent surveys from 2005 to 2008, found that the unprompted recall of graphic cigarette packet warnings increased significantly at each year surveyed (2005, 0.0%; 2006, 14.0%; 2007, 9.0%; and 2008, 12.0%) [23]. However, they also point out that unprompted recall of new graphics and its associated health beliefs is at its peak in the year that the warnings were introduced (2006, 14.0%). Therefore, new information attracted more attention and by extension, promoted better recall than old information, as illustrated by the decline in recall to 9.0% in 2007. These findings contradict the effect of the familiarity bias (*i.e.*, familiar information is easily recalled).

In comparison, the use of symbols or graphics in medication information leaflets did not enhance recall in a low health literate study population [24]. Participants were from Jackson, Tennessee, USA and were recruited from the local literacy council and basic education programs for adults. In order to assess the general literacy of potential participants, they were administered the Rapid Estimate of Adult Literacy in Medicine (REALM) test. The REALM is a measure for assessing reading ability by testing the pronunciation of medical words. This study was performed using an interviewer-administered questionnaire, whereby each participant was given one minute to review a leaflet and then questioned on their recall of the information. Despite the author’s hypothesis that the inclusion of symbols would generate better recall in low health literate populations, they found that the symbols did not enhance short-term recall of information. A limitation in this study could be that the sample may not have been a “low literate sample” since participants were able to read warning information. Another possible limitation in this study could be that the symbols that were used were not understandable. Therefore, the role of symbols on information recall is likely to depend on the comprehensibility of the pictogram.

### 2.4. Studies on the Recall of Warning Information among Workers

As for comprehension, the recall of label elements among workers in SA was found to be low as only three out of 12 warning symbols were found to have more than 50% recalled among workers, namely, skull and crossbones, flammable and explosive symbols [6,20].

Laughery (2006) has identified design and non-design factors important for the effectiveness of warning instruments such as labels [1]. Design factors include size, location, color, signal word and the use of pictorials, whereas non-design factors relate to the target audience and the specific context of the warning information. According to Wogalter *et al.*, 2002, the most important factor for hazard information to be effective is that a warning needs to be clear and noticeable [18]. Several guidelines have been developed for assessing comprehension of symbols/pictograms. In this regard, the presence of pictorials improved the recall of warnings. The studies conducted in South Africa and Malaysia on workers have found that the pictogram was the most frequently recalled element on the label after giving it to the subject for one minute and then withdrawing it [6,19]. These two studies used the GHS pictograms.

In a study of 54 Turkish military pilots, it was found that when symbols were included on warnings used in flight manuals, the symbols contributed to the effectiveness of a warning [25]. This was established by a test whereby participants were asked to match a designed symbol to a warning message. In the latter study, the comprehension levels of the skull and crossbones symbol and the plane with a broken wing symbol were high (>85.0%). The fact that the participants were military pilots, well-educated and aged 24–38 years, may have accounted for the high level of comprehension. In another study on the inclusion of pictograms in warnings, Boelhouwer *et al.* (2013) administered questionnaires to 90 undergraduate students from Auburn University, USA (non chemical users) and 45 members of selected professional societies including the Society for Chemical Hazard Communication, the American Industrial Hygiene Association, and the American Society of Safety Engineers (expert chemical users) [13]. Two versions of a SDS were created for two unnamed chemicals—one with GHS pictograms plus text and one with text only. On separate occasions, participants were asked to answer questions regarding both versions of the SDS. They found that the inclusion of pictograms on the SDS significantly decreased the time to respond to the questions in both the non-chemical users and expert chemical users. However, all the participants in this study were literate implying that they were able to read the text regardless of the pictogram. In contrast, Rother (2008) found that Western Cape farm workers relied on their cultural and socio-economic background to interpret FAO pesticide pictograms on pesticide labels [14]. The meanings they attributed to the pictograms were not linked to the intended definition but were rather from their environment due to their lack of training. Similarly, a study of 31 trade and industry workers selected from a marketplace in Accra-Tema, Ghana examined the comprehension of symbols, which are commonly used in the USA [26]. The symbols were tested without an attached context by asking participants what they meant. Only two out of the six symbols elicited more than 50.0% correct responses, namely, skull and crossbones (81.0%) and prohibition (58.0%). This highlights the difficulty in cross-cultural interpretations of symbols and the non-design factor (*i.e.*, context), which is crucial in designing warning information. Nevertheless, these studies demonstrate that pictograms is a design factor which appears to be vital for the comprehension and recall of hazard information, taking into consideration the context in which the information will be accessed.

The level of education, gender and age has also been examined with respect to the recall of warning information. A meta-analysis of 48 studies, conducted between the years 1975 and 2001, on the effectiveness of warning labels found that recall was not correlated with age [27]. However, the studies in the meta-analysis were conducted on participants aged in their mid-thirties to forties so data on the effect of older age on recall is lacking. In addition, details on the countries in which these studies were done were not provided.

## 3. Conclusions

This review has demonstrated that the levels of comprehension and recall among consumers and workers of hazard information are generally low although some symbols such as the skull and crossbones symbol and flammable symbol are generally well recalled and comprehended. For workers, appropriate training in the correct interpretation of GHS hazard and precautionary information on the label and SDS is an important factor to improving comprehension, and more importantly application, of this information. The evidence on the effect of training on comprehension and recall is, however, limited. Color blindness and demographic characteristics such as age, gender and level of education have been identified as additional factors that could influence comprehension and recall among consumers and workers but the evidence is inconsistent. The effect of demographic factors on recall is important to consider as they are markers of actual determinants of the comprehension and recall of hazard information, for example, gender and age effects might be due to novelty of stimuli and experience. Non-design factors such as the target audience and design factors such as size, location, color, signal word and the use of pictorials are important for the effectiveness of warning instruments such as labels.

There is minimal literature on the recall of chemical hazard information, especially on the factors determining effectiveness of chemical hazard information. Previous studies have found generally inconsistent results on the effect of education, gender and age among consumers and there are hardly data among workers. As stated before, the data on the effect of training on workers is limited. Therefore studies investigating the role of speaking and reading ability, vision, occupation, education, training, gender and age on recall of hazard information are required. Further investigation of the effect of design and non-design factors on recall should be examined as well as studies from different regions of the world to compare contextual differences. Further studies are also needed to estimate the effect of training on the comprehension and recall of hazard information in low literate populations of chemical users (e.g., domestic workers), as is more common in LMIC. In addition, other predictors of recall need to be identified in order to determine strategies to improve the recall of hazard information.

With a better understanding of the factors impacting on recall of chemical hazard information, there is the potential to improve on how hazard information is presented to consumers and used in workers’ training. This in turn would improve the understanding and recall of workers and other users, particularly with low literacy levels, in regard to the chemical hazards they are exposed to which should improve their health and safety decision-making to reduce exposure risks when using the chemicals.

## Figures and Tables

**Table 1:** Summary of studies investigating comprehension and/or recall of health and safety information.

Authors	Study Design and Sample	Measurement of Comprehension/Recall	Predictor	Findings
Dalvie, M.A., Rother, H. and London, L. 2014 [6]	South African study on 402 consumers and workers in four target sectors (agricultural, industrial, transport and consumer) in 2003	Comprehension measured using the hazard communication comprehensibility testing tool		Skull and crossbones (98.0%) and flammable (93.0%) symbols were well understood whereas the least understood were the corrosive and compressed gas symbols (>5.0%)
Houts *et al.*, 2006 [8]	Review of studies in health education, psychology, education and marketing journals on the role of pictures in improving health communication	Comprehension and recall		Pictures increased the recall and comprehension of health education information
Adane, L. and Abeje, A. 2012 [11]	Chemistry and biology (*n* = 83) undergraduate students at Jimma University in Ethiopia (2011)	Comprehension measured using questionnaires	Familiarity	The majority (56.8%) were not familiar with hazard warning signs of laboratory chemicals Low comprehensibility was associated with the need for training
Banda, S.F. and Sichilongo, K. 2006 [12]	Survey of 364 participants in four target sectors (agricultural, industrial, transport and consumer) in Zambia	Comprehension was measured by respondents ranking the label elements in the order of the most danger implied (for example, harmful-warning-caution-attention)	Education Gender Age	Level of education, gender and age was not associated with the level comprehension of GHS label elements
Boelhouwer, E., Davis, J., Franco-Watkins, A., Dorris, N. and Lungu, C. 2013 [13]	90 undergraduate students from Auburn University (naive chemical users) and 45 members of selected professional societies including the Society for Chemical Hazard Communication, the American Industrial Hygiene Association, and the American Society of Safety Engineers (expert chemical users)	Responses were measured using a questionnaire		Inclusion of pictograms on SDS significantly decreased the time to respond to the questions in both the naiveand expert chemical users
Rother, H., 2008 [14]	South African study of 115 farm workers in the Western Cape	Comprehension	Gender	Of the ten pictograms examined, only one was found to have more than 50.0% correct responses, namely, wear gloves (74.8%). males had more correct responses than females
Lehto, M.R. 1998 [15]	111 engineering students from Purdue University in the United States of America (USA)	Comprehension measured using survey instrument and scored using a rating scale varying between 1 and 5. A rating scale of 1–5 was also used to score ease of finding information		Comprehension of labels was correlated with the ease of finding the information on the label (*r* = 0.71)
Lesch, M.F. 2003 [16]	92 participants from the USA recruited through local newspapers	Comprehension—investigated the impact of training methods on the comprehension of symbols	Age	Dramatically improved comprehension especially among the younger participants aged between 18 and 35 years (88.0% correct)
Lesch, M.F. 2008 [17]	43 participants from the USA recruited through local newspapers	Comprehension—investigated the impact of training methods on the comprehension of symbols	Training	Training improved comprehension—verbal training improved comprehension by 30% and accident scenario training improved comprehension by 36%
Ta, G.C., Mokhtar, M.B., Mohd Mokhtar, Hj Anuar Bin, Ismail, A.B. and Abu Yazid, Mohd Fadhil Bin Hj 2010 [19]	150 Malaysian industrial workers	Comprehension measured using the hazard communication comprehensibility testing tool	Tertiary Education Position in the workplace Age Gender	A difference in the comprehension of GHS label symbols with the flammable symbol (99.3%) well understood and the compressed gas (27.3%) poorly understood Tertiary level education improved the comprehension of GHS symbols Higher position in the workplace led to a better comprehension of GHS symbols, whereas gender and age did not contribute to the comprehension of symbols
Andreeva, T.I. and Krasovsky, K.S. 2011 [21]	Nationwide survey of 2000 randomly selected Ukrainian adults	Recall was measured by asking the participants to describe the warnings	Tertiary Education Age Gender	People who completed a higher level of education recalled more warnings Multivariate analysis using linear regression showed that recall declined significantly (*p* < 0.05) with increase in age and males were more likely to recall warnings (*p* < 0.05)
Miller, C.L., Quester, P.G., Hill, D.J. and Hiller, J.E. 2011 [23]	Australian smokers who were interviewed in four independent surveys from 2005 to 2008	Recall measured by using a questionnaire		Recall of graphic cigarette packet warnings increased significantly at each year surveyed (2005, 0.0%; 2006, 14.0%; 2007, 9.0%; and 2008, 12.0%)
King, S.R., McCaffrey, D.J., 3rd, Bentley, J.P., Bouldin, A., Hallam, J. and Wilkin, N.E. 2012 [24]	Participants were from Jackson, Tennessee, USA and were recruited from the local literacy council	Recall measured using a questionnaire		The use of symbols or graphics in medication information did not enhance short term recall in a low health literate study population. The mean recall score with text only was 6.54 (SD = 1.40), text with symbols was 6.65 (SD = 1.40)
Erdinc, O. 2010 [25]	54 Turkish military pilots, well educated and aged 24–38 years	Comprehension measured by asking participants to match a designed symbol to a warning message		Symbols contributed to the effectiveness of a warning. comprehension levels of the skull and crossbones symbol and the plane with a broken wing symbol were high (>85.0%)
Smith-Jackson TL, E.A., 2002 [26]	31 trade and industry workers selected from a marketplace in Accra-Tema, Ghana	Comprehension	Age	
Argo, J.J. and Main, K.J. 2004 [27]	Meta-analysis of 48 studies, conducted between the years 1975 and 2001	Recall		Only two out of the six symbols elicited more than 50.0% correct responses, namely, skull (81.0%) and prohibition (58%) Recall was not correlated with age
